# Investigating PSMA differential expression in canine uroepithelial carcinomas to aid disease-based stratification and guide therapeutic selection

**DOI:** 10.1186/s12917-022-03544-6

**Published:** 2022-12-20

**Authors:** Matthew R. Berry, Bahaa A. Fadl-Alla, Jonathan Samuelson, Thomas J. Rosol, Timothy M. Fan

**Affiliations:** 1 Department of Veterinary Clinical Medicine, College of Veterinary Medicine, University of Illinois at Urbana-Champaign, Urbana, IL 61802 USA; 2 Department of Pathobiology, College of Veterinary Medicine, University of Illinois, Urbana, IL 61802 USA; 3 Heritage College of Osteopathic Medicine, Ohio University, Athens, OH 45701 USA; 4 Cancer Center at Illinois, University of Illinois, Urbana, IL 61802 USA

**Keywords:** Comparative oncology, Canine, Urothelial carcinoma, prostate carcinoma, druggable target, Prostate-specific membrane antigen, Chemotherapy

## Abstract

**Background:**

In male dogs, uroepithelial cancers include invasive urothelial carcinoma (iUC) and prostate carcinoma (PCA). The inability to distinguish iUC involving the prostate from PCA results in indiscriminate clinical management strategies that could be suboptimal as first-line chemotherapy for iUC (cisplatin) and PCA (docetaxel) differ in people. Prostate specific membrane antigen (PSMA) is a transmembrane protein, and its overexpression has been identified in human prostate carcinoma and neovasculature associated with solid tumor growth. This study investigates whether differential PSMA expression exists between presumptive canine iUC and PCA among cell lines and archived patient samples, which might allow for improved accuracy in disease-based stratification and optimal chemotherapy selection. Additionally, in vitro sensitivities of reported canine iUC and PCA cell lines to uroepithelial directed chemotherapeutic agents were characterized.

**Results:**

Normalized PSMA gene and protein expressions were not significantly different between 5 iUC and 4 PCA cell lines. PSMA protein expression was uniformly observed in uroepithelial cancers regardless of anatomic origin from archived patient samples, further confirming that PSMA cannot differentiate iUC from PCA. In vitro sensitivity of cell lines to uroepithelial directed chemotherapeutics revealed that vinblastine exerted the broadest cytotoxic activity.

**Conclusions:**

Differential expression of PSMA was not identified between canine iUC and PCA cell lines or archived patient samples, and PSMA alone cannot be used for disease stratification. Nonetheless given its conserved overexpression, PSMA may be a targetable surface marker for both canine iUC and PCA. Lastly, in uroepithelial carcinomas, vinblastine might exert the broadest anticancer activity regardless of cellular origin.

**Supplementary Information:**

The online version contains supplementary material available at 10.1186/s12917-022-03544-6.

## Introduction

Pet dogs with uroepithelial carcinomas such as invasive urothelial carcinoma (iUC) or prostate carcinoma (PCA) commonly present with lower urinary signs including hematuria, dysuria, and stranguria. Although uroepithelial carcinomas can metastasize, locoregional disease progression resulting in urinary obstruction is expected to become life-limiting in the majority of pet dogs. Although surgical treatments and radiation therapy have been explored for managing localized uroepithelial cancer in dogs [1–5], medical management with non-steroidal anti-inflammatory drugs (NSAIDs) and chemotherapy have been mainstay treatments [6–8], yet robust and durable anticancer activities are variable and inconsistent. In part, the marginal cytoreductive effects of conventional cytotoxin approaches could be a consequence of misinformed diagnosis and improved therapeutic outcomes might be achieved through definitive tumor histologic classification. Such is the clinical paradigm in human medicine, whereby chemotherapy treatment for uroepithelial carcinomas is distinct and based on whether the underlying malignancy is either iUC or PCA in adult male cancer patients. First-line chemotherapy for iUC requires cisplatin-based protocols whereas docetaxel is instituted as treatment of choice for men diagnosed with PCA [9–12]. In the absence of histologic distinction and exacerbated by limited evidence-based clinical trials, conventional practices in veterinary oncology are relegated to instituting pan-uroepithelial directed chemotherapeutics including mitoxantrone, carboplatin, or vinblastine. However, the current inability to reliably distinguish PCA from iUC involving the prostate in male dogs may be resulting in suboptimal first-line chemotherapeutic recommendations with consequent inferior treatment outcomes.

Prostate-specific membrane antigen (PSMA) is a transmembrane protein that has been shown to be significantly overexpressed in human prostate carcinoma and to a lesser extent in tumor neovasculature of various solid tumor histologies [13, 14]. Based upon its highly preferential expression profile, PSMA serves as a clinically relevant differentiating cell surface biomarker and druggable target for PCA in men. PSMA overexpression in men with prostate carcinoma has been leveraged in the development of theragnostic PSMA targeting radionuclides, including FDA approved Lu177 Vipivotide Tetraxetan, providing sensitive and specific serial PET monitoring while also improving the management of PSMA positive, hormone refractory PCA [15–18]. Other PSMA-directed therapies also include chemotherapy-conjugated or radiolabeled antibodies and small molecule PSMA inhibitors [15, 19], and are intensively being investigated for clinical translation. In addition to its unique high expression in PCA, PSMA expression has been observed in tumor neovasculature of almost all solid tumors, and not in normal vasculature, supporting the possibility that PSMA-directed therapies can represent a novel anti-angiogenic strategy that may aid in the management of diverse solid tumors [14, 20].

While less studied in veterinary medicine, canine PSMA protein characterization and cross-species reactivity of antibodies to canine tissues have been reported [21–24]. In the era of comparative oncology and given that the dog is the only other known large mammalian species to spontaneously develop prostate cancer [25], there exists scientific justification for investigating PSMA expressions in canine prostate cancer and other solid tumors, with the potential to advance PSMA theragnostic strategies to benefit pet dogs and lay the groundwork for future human applications. One prior investigation showed that PSMA expression by immunohistochemistry was evident in 50% of canine prostate cancer samples (10/20). The study also suggested that canine prostate cancers most likely originates from the collecting ducts rather than from the peripheral acini and that canine prostate cancers most closely resembles human androgen refractory, poorly differentiated prostate cancer [24]. Complementing natural disease expression of PSMA in pet dogs with PCA, implantation of an immortalized canine PCA cell line (DPC-1) into immunosuppressed research dogs validated the feasibility to monitor primary PCA tumor growth and subsequent development of distant metastases using a molecular imaging strategy with SPECT/CT and PSMA-binding antibody as a radiotracer [26]. Taken together, PSMA expression studies in dogs with PCA highlight the potential use of PSMA-directed theragnostics, and through a comparative oncology lens, the dog may help bridge the gap between preclinical modeling and human clinical trials for these technologies.

Given the divergent first-line chemotherapeutic options in managing human uroepithelial cancers as well as the ability to specifically target PCA using PSMA-directed theragnostics in humans, we sought to determine if PSMA could differentiate between canine iUC and PCA using heavily referenced cell lines and archived patient tissues. Overarching goals of this investigation were to direct the practice of veterinary oncology towards a more customized chemotherapeutic selection process, as well as provide foundational data to justify future exploratory studies evaluating PSMA-targeted approaches for canine uroepithelial cancers and other solid tumors.

## Materials and methods

### Cell lines

Human PCA cell lines (LNCaP and PC-3) utilized as controls were purchased from ATCC (Manassas, VA). Canine iUC cell lines (K9TCC, AxA, AxC, In, and Nk) were generously provided by Deborah Knapp (Purdue University). Canine PCA cell lines (ACE-1, Leo, Probosco) were generously provided by Tom Rosol (Ohio University) and CPA was generous provided by Monique Doré (Université de Montréal). Cell lines were cultured in Dulbecco’s modified eagle medium (DMEM, Corning 10–013-CV)) supplemented with 10% fetal bovine serum (FBS, Gemini 900–108) and 1% penicillin/streptomycin (Lonza 17-602E) and were grown in standard conditions (37 °C in a humidified 5% CO_2_ atmosphere).

### Quantitative PCR

RNA was isolated using a commercially available kit (Qiagen, #74104). The concentration and purity of RNA (assessed by A260/A280 and confirmed to be 1.9–2.0 for all samples) was determined using a NanoDrop1000 spectrophotometer (Thermo-Fischer, Waltham, MA). 1 *μ*g RNA was reverse transcribed using Superscript III First-Strand kit (Invitrogen, #18080–051) and the resultant cDNA was diluted 1:10 in sterile water to a final volume of 200 μL. 5 *μ*L of cDNA was used as a template in a 25 *μ*L PCR also containing 1.25 *μ*L canine specific TaqMan PSMA primer (Cf02738537_g1) or GAPDH primer (Cf04419463_gH) and 1X TaqMan Fast Advanced Master Mix (Thermo Fisher, 4444557). All quantitative PCR reactions were performed in a 96-well reaction plate (Thermo-Fisher, N8010560), performed in two biologic replicates, and completed using the Applied Biosystems 7500 Real-time PCR Thermal cycler (Thermo-Fisher, Waltham, MA).

### Cell lysates and western blot analysis

Cell pellets were lysed on ice in Mammalian Protein Extraction Reagent (MPER, Thermo Fisher, 78,503) containing Halt Protease Inhibitor Cocktail (Thermo Fisher, 87,786). Lysates were quantified using the Pierce BCA Protein Assay kit (Thermo Fisher 23,225). 10 μg of LNCaP and 50 μg other PCA cell line samples were then denatured (95 °C for 5 minutes), separated by SDS-PAGE (4–20%), and transferred to a nitrocellulose membrane. Membranes were blocked in 5% non-fat milk, dissolved in tris-buffered saline-tween 20 (TBST), for 1 hour at room temperature prior to primary antibody incubation. PSMA protein expression was evaluated using a mouse monoclonal, anti-human PSMA antibody (Novus Biologicals, NBP1–45057) with demonstrated reactivity to canine PSMA [23]. The PSMA antibody was diluted to 1 *μ*g/mL and membranes were incubated overnight at 4 °C, followed by secondary labelling using species a specific HRP-linked antibody (Cell Signaling) for 1 hour at room temperature. Membranes were then developed using SuperSignal West Femto Maximum Sensitivity Substrate (Thermo Fisher 34,094) and ChemiDoc XRS+ molecular imager system. All membranes were re-probed for *β*-actin (Abcam, ab6276) for use as loading control.

### PSMA immunohistochemistry

Cell lines were fixed in 10% formalin for 1 hour at room temperature. After 1-hour incubation, cells were washed and pelleted in 2% agarose, then paraffin-embedded. The University of Illinois Veterinary Diagnostic Laboratory database was reviewed for cases of iUC among female dogs (for undisputed iUC confirmation) and carcinomas of the prostate. In total, 12 iUC cases and 10 carcinomas of the prostate were identified to be recut for PSMA immunohistochemistry (IHC). IHC staining was performed using an autostainer (intelliPATH FLX, Biocare, Concord, CA). Samples were deparaffinize in xylene and rehydrated in alcohol. Samples were treated with Diva Decloaker and then blocked with Peroxidazed 1 for 5 minutes. A secondary block with Background Punisher was performed for 10 minutes. Samples were incubated with a mouse monoclonal, anti-human PSMA antibody (Novus Biologicals, NBP1–45057) at 1:1000 dilution for 30 minutes. Samples were washed, incubated with a biotinylated secondary antibody for 30 minutes, washed, then incubated with the chromogen IP FLX DAB. Slides were counterstained with hematoxylin. Well characterized human cell lines served as strong positive (LNCaP) and negative/weak positive (PC-3) PSMA controls.

### Cytotoxicity assessment using Sulforhodamine B assay

Cell lines were seeded in 96-well plates, at a concentration of 2000–4000 cells per well (optimized to achieve 100% confluency at 72 hours in untreated/control wells), and allowed to adhere overnight (12–16 hours). Medical grade carboplatin, mitoxantrone, and vinblastine were used in these studies. Docetaxel was purchased (Cayman Chemical, 11,637) and solubilized in DMSO. Treatments ensued for 72 hours with each chemotherapeutic prior to completing SRB assays as previously described [27]. Several smaller cohort cytotoxicity experiments (evaluating a few cell lines at a time) were performed to optimize seeding densities and drug concentration ranges before completing the full cell line cytotoxicity experiment. Based on these findings, the following drug concentrations were selected for the full cell line cytotoxicity experiment: vinblastine (0.01pM-100 μM), docetaxel (10pM-1 μM), mitoxantrone (0.3 nM-30 μM), and carboplatin (0.39 μM–400 μM). Each drug concentration was performed in four replicate wells. Magnitude of chemotherapy-induced cytotoxicity was normalized against untreated control (carboplatin, mitoxantrone, and vinblastine) or DMSO control (docetaxel). Results were graphed using ggplot2 (version 3.3.5) in RStudio (RStudio 2021, Boston, MA), and data grouped based upon cancer of origin (iUC versus PCA).

### Statistical analysis

The half maximal inhibitory concentration (IC50) for each cell line histology classification (iUC versus PCA) following exposure to each chemotherapeutic agent (carboplatin, mitoxantrone, vinblastine, and docetaxel) was extracted using AAT Bioquest IC50 calculator [28] 4 parameter fitting. IC50 values for curves failing the 4 parameter fitting (*n* = 12) were generated with 2 parameter (point-to-point, *n* = 2) or 3 parameter (*n* = 10) fitting. Given that normality was not met using the Shapiro-Wilk test, Wilcoxon rank-sum testing was utilized to determine if IC50 differences existed between iUC and PCA cell lines following 72 hour exposure to each chemotherapeutic agent. RStudio (RStudio 2021, Boston, MA) was used for statistical testing, and significance was defined as *p* < 0.05.

## Results

**Fig. 1:**
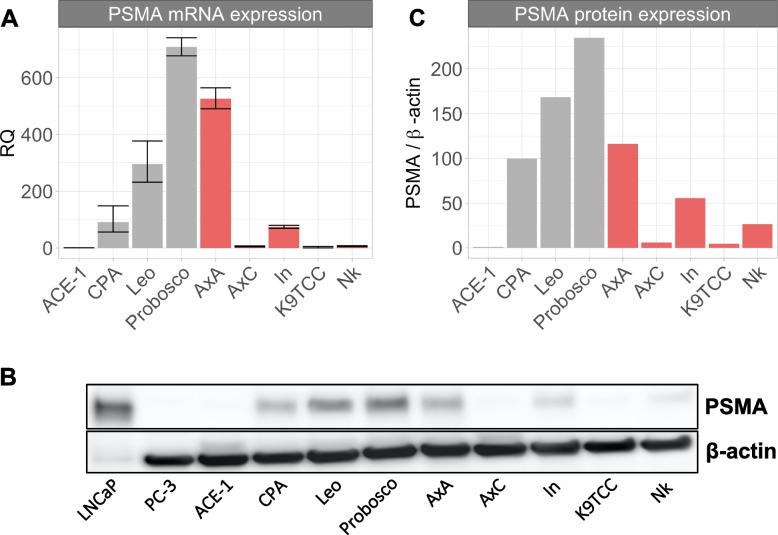
PSMA gene & protein expression among canine uroepithelial carcinoma cell lines. (A) PSMA gene expression patterns among canine uroepithelial carcinoma cell lines. Error bars represent RQ min and max values from a representative biologic replicate. (B) PSMA western blot image with known human control cell lines (LNCaP +, PC3-), and (C) PSMA protein expression normalized to *β*-actin loading control. Within bar graphs, grey represents canine PCA cell lines and red represents canine iUC cell lines

To evaluate whether PSMA may be differentially expressed among canine uroepithelial carcinomas, comparative mRNA and protein expression studies were performed across heavily referenced canine iUC [29] and PCA cell lines [30–33]. PSMA gene and protein expression levels corresponded across and within individual cell lines; however, PSMA expression was variably detected within both iUC and PCA groups (Fig. 1; full-length immunoblots available in supplemental Fig. 1). Variable PSMA expression levels within each group suggested that PSMA alone could not be used for differential disease stratification.

**Fig. 2:**
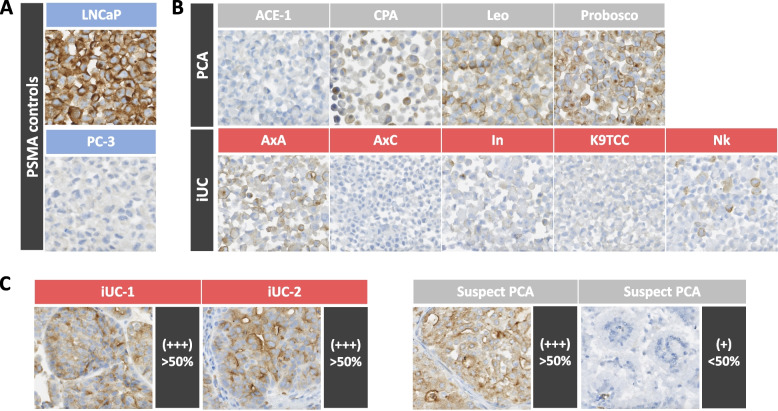
Immunohistochemical detection of PSMA among canine uroepithelial carcinoma cell lines and representative patient samples. (A) PSMA IHC of known human reference control cell lines (LNCaP+, PC3-). (B) PSMA IHC among canine uroepithelial cell lines with grey labeled (top row) representing PCA cell lines and red labelled (bottom row) representing iUC cell lines. (C) PSMA IHC of archived iUC patient sample (red labelled, left) and carcinomas of the prostate (grey labelled, right)

Next, PSMA expression was characterized in archived iUC samples from female dogs, and carcinomas of the prostate from male dogs. PSMA expression was evident among all patient tissue samples and PSMA staining localized to tumor cells, further confirming PSMA could not solely stratify iUC and PCA in naturally occurring tumor specimens (Table 1 and Fig. 2). Intriguingly, the three lowest PSMA expression patterns were from carcinomas of the prostate that were histologically supported to be PCA based on morphology and negative uroplakin-3 expression (data not shown). Collectively, these findings derived from cell lines and naturally occurring tumor samples suggested broader PSMA expression profiles across two distinct uroepithelial cancers commonly diagnosed in dogs, and exclusive PSMA expression in PCA was not supported. The positive PSMA expression patterns noted among evaluated canine uroepithelial carcinoma samples suggested that PSMA could not be used as a discriminatory marker to distinguish iUC and PCA, however, PSMA-targeting strategies could be considered more broadly applicable to uroepithelial carcinomas regardless of cellular origin in dogs.

**Table 1:** Patient sample PSMA IHC

iUC from female dogs (*n* = 12)	Carcinomas of the prostate (*n* = 10)
12/12 (100%) PSMA+	10/10 (100%) PSMA+
9/12 (+++) 3/12 (++)	5/10 (+++) 2/10 (++) 3/10 (+)

*Strong, > 50% positive cells (+++)*

*Weak, > 50% positive cells (++)*

*Weak, < 50% positive cells (+)*

**Fig. 3:**
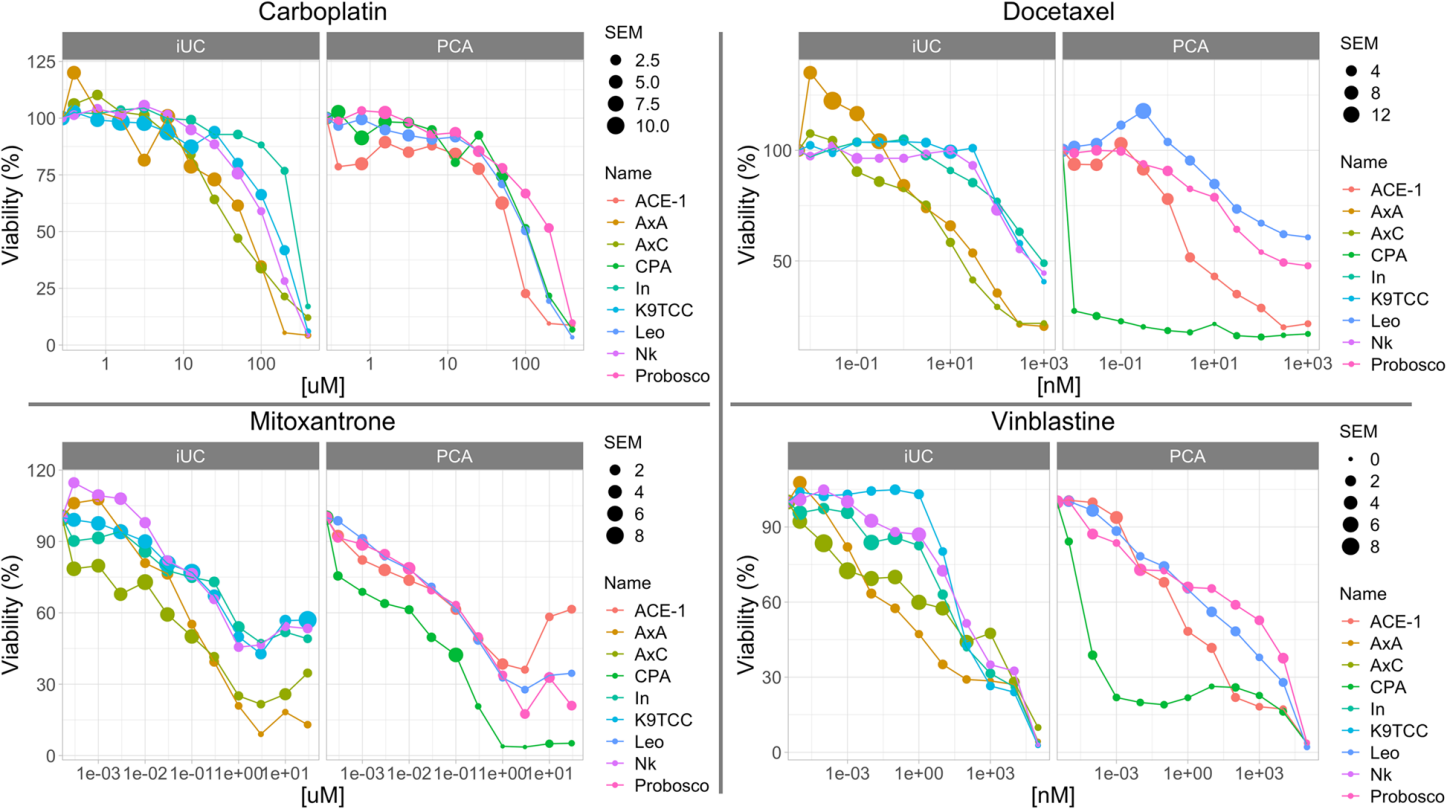
Dose-response line graphs of canine uroepithelial carcinoma cell lines to each urothelial directed chemotherapeutic agent. In each of four panels of different chemotherapeutic agents, 5 canine iUC (left) and 4 canine PCA (right) cell lines were incubated with varying concentrations of drug for 72 hours, then processed using Sulforhodamine B assay. Dot size indicates SEM

To determine if differences in sensitivity among canine iUCs and PCAs to traditional cytotoxic drugs existed in vitro, common uroepithelial-directed chemotherapeutics (mitoxantrone, carboplatin, and vinblastine) as well as docetaxel, the first-line chemotherapeutic for human PCA, were evaluated in cell culture. Figure 3 shows cytotoxic activity generated among the 9 cell lines following exposure to these four chemotherapeutic agents, each having a plot for iUC and PCA cell lines. The drug concentration ranges utilized were of biologic relevance based upon known Cmax pharmacokinetic data in dogs for docetaxel (~ 500 nM) [34, 35], mitoxantrone (~ 0.2 nM) [36], and vinblastine (range of ~ 12-30 nM) [37] whereby predicted Cmax concentrations fall within the upper half of the tested ranges. The carboplatin concentration range was largely determined by in vitro screening sensitivities, and the known Cmax value (~ 0.5 nM) falls below the vitro tested concentration range [36]. The IC50 averages and *p*-values calculated for each chemotherapeutic agent were carboplatin (iUC 146.97 μM, PCA 116.82 μM; p-value = 0.73); docetaxel (iUC 107.08 nM, PCA 7 nM; p-value = 0.19); mitoxantrone (iUC 0.08 μM, PCA 0.05 μM; p-value = 0.389); and vinblastine (iUC 71.61 nM, PCA 57.33 nM; p-value = 0.73) (see Supplemental Fig. 2 for individual IC50 determinations). There were no significant differences in sensitivities across the tested cell lines and chemotherapeutics.

## Discussion

Although surgery can be utilized in select cases, medical management (NSAIDs and chemotherapy) alone or in combination with radiotherapy will continue to play an important role in managing uroepithelial carcinomas in dogs. Uroepithelial carcinomas in male dogs can be difficult to discern, and first-line chemotherapeutic selection may be suboptimal as iUC and PCA are treated differently in humans. This study sought to evaluate PSMA expression among a panel of canine uroepithelial cell lines and archived patient samples to ascertain whether differential expression exists to help distinguish PCA from iUC involving the prostate. The data generated revealed that PSMA is variably expressed among canine iUC and suspected PCA samples, indicating that PSMA expression cannot be used as a sole distinguishing marker for disease stratification.

While there has been longstanding and recognized value of PSMA for the diagnosis and therapeutic management of PCA in people, it has been debatable if PSMA is uniformly and overexpressed in urothelial carcinomas of the bladder in human beings [38]. However, recent studies have emerged detailing the potential prognostic and diagnostic utility of PSMA expressions in urothelial carcinoma [39–42]. Notably, in a large study evaluating urothelial carcinomas in humans, PSMA expression was identified in 78.7% (70/89) of patient samples and was negatively correlated with clinical stage and histologic tumor grade. Considering recent human studies, the identification of PSMA expressions in both iUC and PCA in canine uroepithelial carcinomas is congruent with notion that PSMA is not exclusively restricted to PCA. While insufficient to serve as a differentiating marker, the high and relative conserved expression of PSMA across tumor samples of both iUC and PCA etiology underscores that PSMA-targeting strategies may benefit dogs diagnosed with uroepithelial carcinomas regardless of anatomic origin.

The findings from the current investigation also highlights the potential utility of pet dogs with naturally occurring cancers to serve as a pre-clinical model for PSMA-targeting strategies that can aid disease diagnosis and selective payload delivery of anticancer drugs, small molecule inhibitors, or radionuclides. While limited in number, there have been recent scientific investigations demonstrating the potential value of PSMA expressions in canine hemangiosarcoma (cHSA), a highly vascular solid tumor expected to express PSMA. First, the prognostic value of three immunohistochemical markers, being claudin-5, Ki67, and PSMA were evaluated in 53 splenic cHSA samples with linked clinical outcome data. From this investigation, low PSMA gene and protein expressions correlated with longer survival times [43]. Second, as a model of malignant neovasculature, cHSA cells were shown to express up to 6-fold greater PSMA at both gene and protein levels compared to a normal endothelial cell line. This differential expression of PSMA by malignantly transformed endothelial cells was leveraged to support the diagnosis of cHSA cells within resected tissue samples and hemorrhagic effusions through conventional and molecular diagnostic techniques including immunohistochemistry and polymerase chain reaction, respectively [23]. Last, complementing the prognostic and diagnostic values of PSMA expressions in cHSA, the therapeutic druggability of PSMA has been described in which doxorubicin-loaded nanoparticles decorated with the A10 aptamer were shown to preferentially bind to surface PSMA expressed by cHSA cells, and exerted provocative activity in a heterotopic cHSA mouse model [44]. Additionally, using two canine PCA cell lines (ACE-1 and Leo), canine PSMA has been shown to exhibit similar enzymatic characteristics to human PSMA and can be similarly targeted using small molecule enzyme inhibitors. The gene and protein expressions of ACE-1 and Leo cell lines in our study are congruent with this earlier report [45]. Collectively, these contemporary investigations underscore the potential value of PSMA theragnostic approaches for improving disease diagnosis and prognosis, as well as the feasibility for targeted drug delivery strategies in pet dogs with PSMA-expressing cancers.

Given the distinction in first-line chemotherapies for treatment of uroepithelial carcinomas in human beings, another aim of the current investigation was to characterize in vitro sensitivity to conventional uroepithelial directed chemotherapeutic agents, hypothesizing that iUC and PCA cell lines would have divergent sensitivities that could help clinicians select top choice chemotherapeutics if PCA could be differentiated from iUC involving the prostate. Interestingly, no obvious contrasting sensitivities of iUC and PCA cell lines to mitoxantrone, carboplatin, vinblastine, or docetaxel were identified. Of particular note, PCA cell lines did not appear to be more sensitive to docetaxel compared to iUC cell lines. These cell culture findings are not incongruent with recently described activity of docetaxel in dogs with epithelial malignancies [46]. In this phase II study, 51 dogs were treated with docetaxel with an objective response rate of 16.7% (8/48 partial responses), however only ~ 20% of the evaluated patient population had uroepithelial carcinomas (8 bladder and 2 prostate) and clinical response rates were not different between these 2 limited subpopulations.

Acknowledging the recognized limitations and predictive value of in vitro assays, and guided by the in vitro chemosensitivity data generated in this study, when in doubt clinically, clinicians should consider commonly utilized vinblastine or mitoxantrone as broadly active agents for either iUC or PCA, based upon their capacity to induce cytotoxicity at biologically achievable concentrations across the cell lines tested. While docetaxel also induced cytotoxicity at biologically achievable concentrations in vitro, additional clinical investigations of docetaxel for the treatment of canine urothelial carcinomas are recommended before it may be considered as a first-line therapy in dogs. Carboplatin may also be considered in dogs with newly diagnosed urothelial carcinomas given that it has proven clinical activity as a first-line chemotherapeutic in dogs with urothelial carcinomas [6]. It would be anticipated with more sophisticated in vitro modeling algorithms, chemotherapy response predictions could become more accurate and clinically actionable for optimizing treatment of canine uroepithelial carcinomas. Such is the paradigm currently being tested for canine lymphoma, whereby large data sets inclusive of clinical, molecular, and biologic data can be combined and analyzed with machine learning models to theoretically design optimal chemotherapy protocols [47].

## Conclusion

In summary, PSMA remains a highly promising biomarker for specific tumor types in human beings, and there is gradual exploration of its theragnostic value in veterinary oncology. While PSMA cannot reliably distinguish canine PCA from iUC, its broad expression by uroepithelial carcinomas keeps the door open for future research studies related to PSMA-targeting strategies that include molecular imaging and small molecule inhibitors. Additionally, given no obvious contrasting chemosensitivity profiles between canine PCA and iUC cell lines to common chemotherapeutics targeting uroepithelial cancers, until larger evidence-based clinical trials can be conducted to advise best therapeutic practices for particular uroepithelial cancers in pet dogs, current recommendations for optimal cytotoxin drug selection should be guided primarily by real-time assessment of patient tolerability and objective responses.

## Supplementary Information

**Additional file 1.**

**Additional file 2.**

## Data Availability

The research data generated and analyzed during this study are included in this published article.
