# Supplementary material for: Investigating PSMA differential expression in canine uroepithelial carcinomas to aid disease-based stratification and guide therapeutic selection
Source: BMC Vet Res. 2022 Dec 20;18:441. doi: 10.1186/s12917-022-03544-6 (PMC9764509; doi:10.1186/s12917-022-03544-6)
Supplement: Supplementary file 1 — Additional file 1. [file 12917_2022_3544_MOESM1_ESM.pdf]

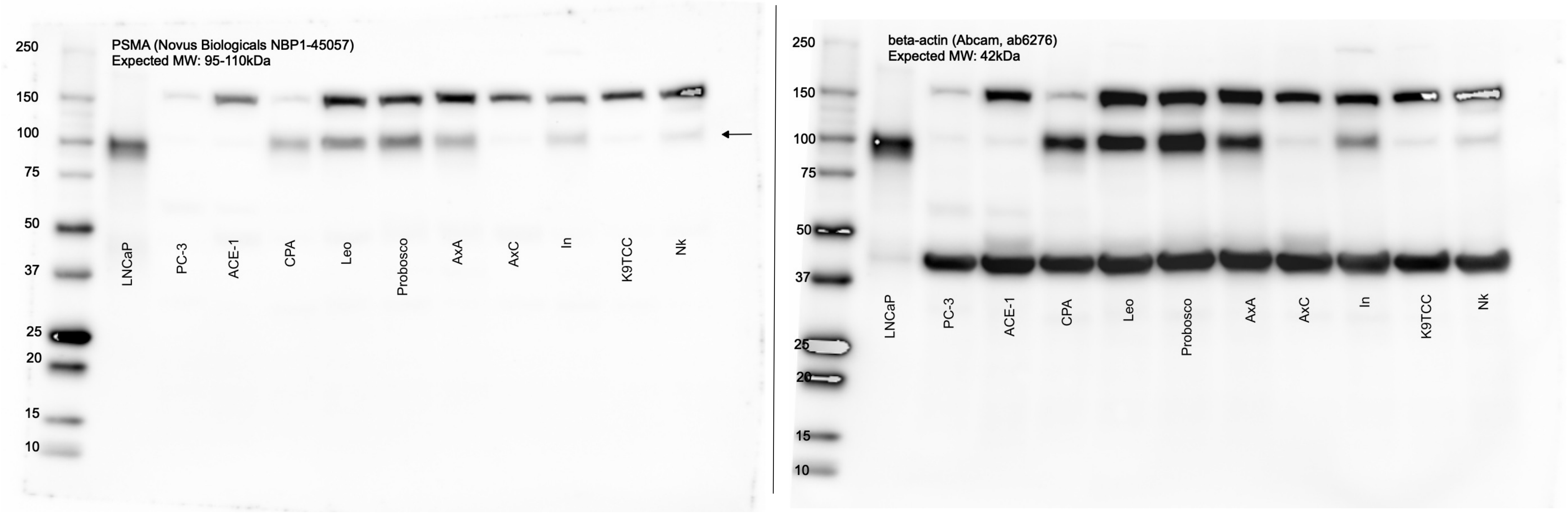

Supplemental Figure 1: Annotated, but otherwise unmodified, full-length immunoblots for PSMA (left) and β-actin (right). All lanes contained 50µg of protein except for LNCaP, which was loaded with 10µg.
