# Supplementary material for: Investigating PSMA differential expression in canine uroepithelial carcinomas to aid disease-based stratification and guide therapeutic selection
Source: BMC Vet Res. 2022 Dec 20;18:441. doi: 10.1186/s12917-022-03544-6 (PMC9764509; doi:10.1186/s12917-022-03544-6)
Supplement: Supplementary file 2 — Additional file 2. [file 12917_2022_3544_MOESM2_ESM.pdf]

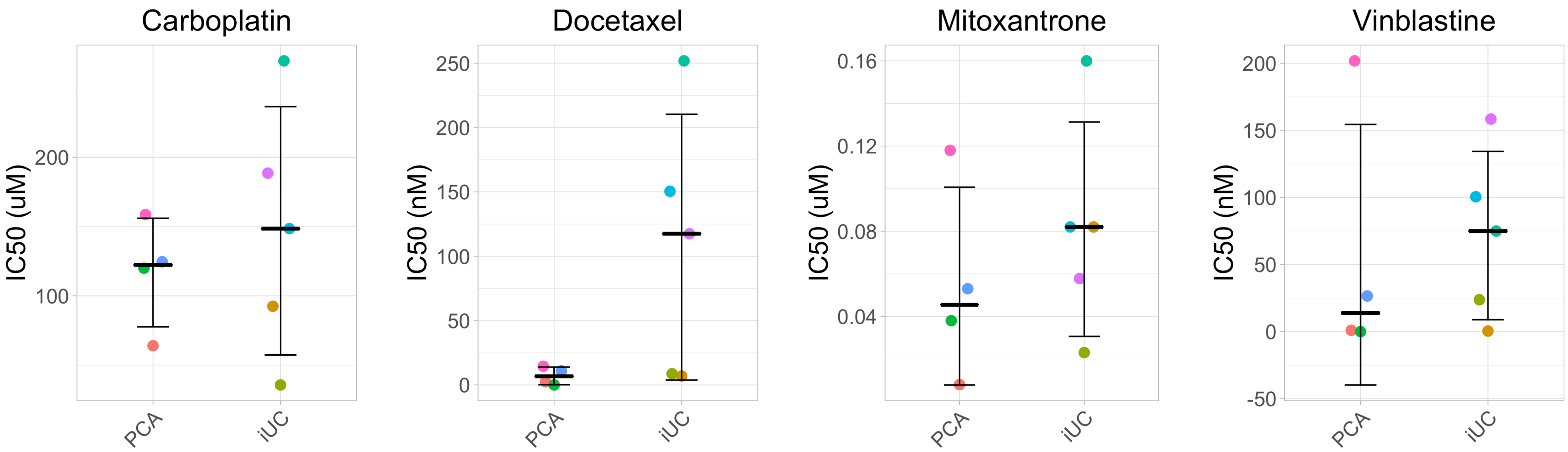

	Carboplatin PCA	Carboplatin iUC	Docetaxel PCA	Docetaxel iUC	Mitoxantrone PCA	Mitoxantrone iUC	Vinblastine PCA	Vinblastine iUC
**ACE-1**	**64.06**		**2.5433**		**0.008**		**1.011**	
**CPA**	**119.98**		**0.006**		**0.038**		**0.00004**	
**Leo**	**124.61**		**10.95**		**0.053**		**26.56**	
**Probosco**	**158.64**		**14.51**		**0.118**		**201.74**	
**AxA**		**92.51**		**6.96**		**0.082**		**0.4232**
**AxC**		**35.74**		**8.7**		**0.023**		**23.77**
**In**		**269.54**		**251.76**		**0.16**		**74.97**
**K9TCC**		**148.5**		**150.46**		**0.082**		**100.5**
**Nk**		**188.57**		**117.51**		**0.0578**		**158.41**

Supplemental Figure 2: IC50 boxplots with IC50 values of individual cell lines exposed to each urothelial directed chemotherapeutic agent. Cells were incubated with varying concentrations of drug for 72 hours, processed by SRB assay, then IC50 values were determined using AAT Bioquest IC50 calculator.
